# Agenda-setting of tobacco control policy in Iran: a retrospective policy analysis study

**DOI:** 10.1186/s12889-021-12339-7

**Published:** 2021-12-15

**Authors:** Hamid Ravaghi, Sogand Tourani, Rahim Khodayari-Zarnaq, Baharak Aghapour, Azita Pishgoo, Jalal Arabloo

**Affiliations:** 1 School of Health Management & Information Sciences, Iran University of Medical Sciences, Tehran, Iran; 2 Department of Health policy and Management, School of Management and Medical Informatics, Tabriz University of Medical Sciences, Tabriz, Iran; 3 Tabriz Health Services Management Research Center, Health Management and Safety Promotion Research Institute, Tabriz University of Medical Sciences, Tabriz, Iran; 4 Student Research Committee, Tabriz University of Medical Sciences, Tabriz, Iran; 5 Department of Community Nutrition, School of Nutrition and Food Science, Tabriz University of Medical Sciences, Tabriz, Iran; 6 School of Public Health and safety, Shahid Beheshti University of Medical Sciences, Tehran, Iran; 7 Health Management and Economics Research Center, Iran University of Medical Sciences, Tehran, Iran; 8 School of Public Health, Tehran University of Medical Sciences, Tehran, Iran

**Keywords:** Health policy, Agenda-setting, Tobacco control, Multiple streams framework, Iran

## Abstract

**Background:**

The prevalence of tobacco use, especially hookah, has increased in Iran In recent years, particularly among young people and women, and the age of onset of use has decreased. Tobacco use is the fourth leading risk factor for non-communicable diseases in Iran. These issues cause concerns in the country and led to the present study on tobacco control agenda-setting in Iran over a 30-year timeframe.

**Methods:**

We conducted this retrospective analytical study to investigate process analysis in Iran using Kingdon’s multiple-streams framework (MSF). We collected the data using semi-structured interviews with key informants (*n* = 36) and reviewing policy documents (*n* > 100). Then, we analyzed the policy documents and in-depth interviews using the document and framework analysis method. We used MAXQDA 11 software to classify and analyze the data.

**Results:**

Iran’s accession to the Framework Convention on Tobacco Control (FCTC) opened a window of opportunity for tobacco control. The policy window opens when all three streams have already been developed. The adoption of the comprehensive law on the national control and campaign against tobacco in the Islamic Consultative Assembly in 2006 is a turning point in tobacco control activities in Iran.

**Conclusions:**

The tobacco control agenda-setting process in Iran was broadly consistent with MSF. The FCTC strengthened the comprehensive plan for national control of tobacco as a policy stream. However, there are several challenges in developing effective policies for tobacco control in the Iranian setting.

## Background

Tobacco control is one of the public health priorities, and the tobacco epidemic is one of the greatest public health threats the world has ever faced. Further, smoking is the leading cause of preventable death in the world [1, 2]. Globally, tobacco accounted for 8.71 million deaths and was the second-leading risk factor for deaths and the third-leading risk factor for disability-adjusted life years (DALYs) in 2019. In Iran, tobacco was the fourth-leading risk factor for attributable DALYs after high blood pressure, high body-mass index, and high fasting plasma glucose in 2019 [3]. The economic costs of smoking are significant, causing hundreds of billions of dollars in economic losses each year worldwide. According to the World Health Organization (WHO), two to three times the cost of smoking is used to treat diseases caused by tobacco use. Besides the high public health costs of treating tobacco-related diseases, smokers are less productive due to the increased incidence of the disease and those who die prematurely deprive their families of much-needed income. Tobacco use and poverty are inextricably linked. Tobacco use increases poverty and prevents economic development [4].

Studies showed that tobacco consumption is increasing in Iran, especially among women, and the age of onset is decreasing. The first global and international tobacco control efforts date back to 1987. Since 1998, the Tobacco Control Program has been at the forefront of the WHO to reduce the global burden of deaths and diseases related to tobacco. A working group was formed to formulate the Tobacco Control Framework Treaty, which in 2003 led to the ratification of the framework convention on tobacco control (FCTC) [5]. Iran also joined the FCTC in 2005. Achieving the effectiveness of policies requires policy changes that arise from the agenda-setting stage, and the purpose of this study was to review and analyze these cases. On the other hand, this study aimed to identify three streams and examine how policymakers placed tobacco control as a political priority in the agenda-setting in Iran over a 30-year timeframe.

### Conceptual framework

We applied Kingdon’s multiple-streams framework (MSF), one of the most commonly used agenda-setting models, as a framework for analysis [6]. Agenda-setting is the first stage of the policy-making process. According to MSF, when the three problem streams, policy streams, and political streams exist, policymakers consider the issue, and it moves onto the agenda [7, 8]. The Problem Streams involve convincing policymakers to pay special attention to one issue due to various statistical indicators, political reports, and pressure from advocacy groups. The Policy Stream involves many ideas competing for acceptance, and a policy solution emerges to solve the problem in this condition. The chances of accepting ideas will increase technical feasibility, acceptance of values, and are in line with the prevailing ideological streams. Political Stream indicated macro-level political situations affected by national mood, changing public opinion, pressure group campaigns and managerial/legal changes [9–12].

This framework assumes that when these three streams come together at critical time points, a “window of opportunity” will appear. When a window opens, policy entrepreneurs should seize the opportunity immediately, take steps to link problems to solutions and perform advocacy of politicians who are receptive to their ideas [10, 13].

## Methods

We conducted this retrospective policy analysis using a case study approach based on Kingdon’s agenda-setting framework to examine Iran’s tobacco control policy process over the past three decades (1984–2016). We applied MSF to analyze principle factors influencing the problem stream of tobacco use, the solutions to control tobacco use, and the political events that affect tobacco control policies. We collected qualitative data using semi-structured interviews with key informants (*n* = 36) and reviewing archival policy documents (*n* > 100).

### Document review

We analyzed all available policy documents related to tobacco. These documents mainly included policy documents, laws, regulations and government reports, scientific literature, national studies and newspaper articles and minutes of meetings. Then, we identified the available governmental reports by searching the web pages of the Iranian government agencies, the Ministry of Health and Medical Education (MoHME), the Universities of Medical Sciences, and related research centers.

### Key informant interviews

We conducted semi-structured interviews for key informants and stakeholders by PhD students in health policy at the time of the study. We developed the interview guide based on a conceptual framework and literature search and piloted it before the study began. The research team confirmed its validity. We applied purposive and snowballing sampling to address key informants. The sequence of questions was not the same for all participants and varied depending on the participants’ research stages and answers. We interviewed 33 key informants and continued until the saturation of data. The majority of interviewees were men (*n* = 24). We interviewed some stakeholders for over one session. We conducted 36 interviews with 33 stakeholders, seven of whom were from different levels of the MoHME, 6 participants were academics and researchers, and 5 were anti-tobacco related non-governmental organizations (NGOs). The other 15 participants were from the Ministry of Interior, Ministry of Economic Affairs and Finance, Ministry of Education, Ministry of Culture and Islamic Guidance, Ministry of Industry, Mine and Trade, headquarters for combating the smuggling of commodities and foreign exchange, Parliament, Municipality of Tehran, Islamic Republic of Iran Broadcasting (IRIB). These interviews took place between 2016 and 2018. Before starting interviews, the researcher performed communication techniques, explaining the research process and ensuring that information would be kept confidential. Then, we started the interviews with an open question and tried to guide the interview process to cover the research objectives by using interview techniques. The interviews lasted between 35 and 65 min, which was an average of 50 min. During the interview, we refused any orientation to the participants’ opinions to ensure that only the participant’s opinion was collected. We performed all interviews face-to-face and recorded them with informed consent. Besides, we asked the following questions about the problem stream and policy stream politics stream:

1. Problem stream

*How, when and by whom did the issue of tobacco enter the government’s agenda?*

2. Policy stream

*What were the solutions suggested by different organizations to address tobacco-related issues in Iran?*

3. Political stream

*What were the political factors that have influenced tobacco policies in Iran? What has been the impact and political communication with stakeholders in the policy-making process?*

### Data analysis

We used the framework analysis to analyze the data based on applying MSF. In the first step, we read the archival documents and transcribed interviews repeatedly to familiarise researchers with data. Then, we categorized data through coding and summarization techniques. Two authors performed open-ending coding to extract themes. Two researchers discussed disagreements to resolve. According to Kingdon’s framework, we extracted themes, including problem identification, policy solutions, and political opportunities. We used MAXQDA 11 software to analyze the data. We conducted the study and reported by consolidated criteria for reporting qualitative research (COREQ) guidelines [14].

### Ethical issues

The Research Ethics Committee of the Tehran University of Medical Sciences (TUMS) approved the study (code 8921557003). We provided the interviewees with an oral explanation of the nature and objectives of the research. All interviewees received information about the research and the consent form before the interview and had the right to withdraw from the study at any stage of the research. We observed the principle of confidentiality of information in the research results and did not state the issues that led to the recognition of the interviewee. We provided the results of the research to the interviewees upon their request.

## Results

**Fig. 1:**
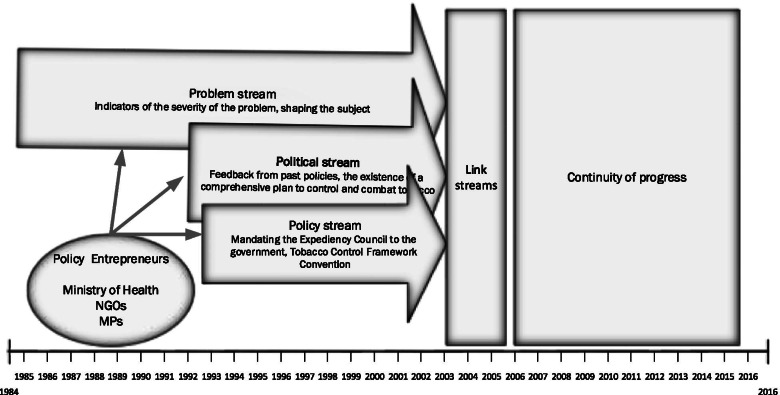
Tobacco agenda-setting in Iran based on the Kingdon’s multiple streams framework

In this section, we will discuss the tobacco control agenda-setting process based on MSF. The findings of this study result from interviews with key informants and documentary analysis (Fig. 1).

### Problem stream

#### Indicators of problem severity

Participants stated that the prevalence of tobacco use has increased in Iran, especially among young adults. It has led to economic losses, which had awakened proponents of tobacco control in the country.

“… 12 to 15 percent of the population, equivalent to 10 to 12 million people in Iran*,* are smokers. Smoking between the ages of 15 and 24 has risen from 10 to 17 percent in the last *ten* years. If every smoker consumes 3000 rials per day for cigarettes, between 30 to 40 billion rials will be smoked daily in the country, which will be close to 10000 to 15000 billion rials per year …” (Participant. 33).

The results of the surveys conducted during this period were in line with the interviewees’ statements. In 1991, the first national study was conducted on the prevalence of tobacco use based on the “National Health and Disease Survey” in Iran. The trend of changes showed that the prevalence of tobacco use has decreased (1991–1999) based on two National Health Surveys (in the age group of 15–69 years from 14.6 to 11.7%). Hookah use decreased from 3.8 to 3.5% and followed a similar pattern in the general population, but increased from 0.8 to 1.4% in the age group of 15–24 years; therefore, the results of the National Health and Disease Survey showed an increase in hookah use in the age group of 15 to 24 years [15, 16]. Several other studies were conducted on the prevalence of tobacco use in the country, such as the Global Youth Tobacco Survey (GYTS) [17], which led to the accumulation of valid information about the severity of the problem in Iran.

Interviewees also stated that the gating hypothesis is one of the most important factors that led to tobacco in Iran. It means that tobacco is considered a gateway to addiction and other social crimes.

The interviewees stated that the economic and health consequences are among the most critical factors that led to tobacco use in Iran. Researchers and advocates of tobacco control have to estimate mortality and morbidity information related to tobacco in Iran based on global evidence and production information, although it did not exist.

Some interviewees highlighted the activities of multinational corporations and cigarette smuggling, which increased the access to people in the community, especially young adults. An examination of the documents indicated that multinational tobacco companies considered Iran a viable market for their products and a gateway for smuggling cigarettes from other countries.

“International tobacco companies planned to enter the markets of developing countries after reducing their sales in the domestic market*”* (Participant. 33).

“*Unfortunately, multinational cigarette companies have chosen Iran as a focal point for promoting and expanding cigarettes for two reasons. The major factor is that Iran is a young population of nearly 40 million people under the age of 30, which is a suitable environment and platform for encouraging smoking. Statistics show that quitting smoking is almost impossible for those who start smoking at a young age. Second, Iran is a safe passage for smuggling cigarettes and tobacco because of its strategic location, at a strategic crossroads between Central Asia and Europe”* [18].

#### The role of religion

After the Islamic Revolution of Iran, a group of experts formed a social-religious movement to combat the consumption of tobacco and cigarette companies. They tried to limit tobacco production, smoking in public places, and the promotion of tobacco and various items of cigarettes. Their efforts led to agenda-setting of tobacco control in the country and the approval of the Fatwa (Islamic Order) on the ban of smoking in the Islamic Consultative Assembly in 1992 as a prelude to the development of other tobacco control policies in the country [19, 20].

#### Framing the issue

Interviewees often referred to the issue of tobacco as a crisis or a social problem. In Iran, during this period, terms were often used to describe the severity of the problem of tobacco use, such as “epidemic”, “scourge”, “social problem”, “social harm” and “colonial gift”.

### Policy stream

This process started in 1991 and developed in the early 2001s as a “Comprehensive Plan on National Control and Campaign against Tobacco” as an acceptable and comprehensive solution and advocate for tobacco control.

#### Feedback from previous policies

The findings of the present study revealed that feedback from past tobacco control policies influenced the policy stream. The interviewees stated that policymakers and actors’ perceptions of the inadequacy, incompleteness, and non-implementation or failure of previous bylaws and directives led to a sense of the need for a comprehensive law. It led to a comprehensive plan to control and combat tobacco use by members of Parliament and then a bill to control tobacco use by the government. Initiatives and plans related to tobacco control as a public health issue go back to 1994 when the tobacco control program was one of the MoHME’s priorities. Therefore, a committee comprising full-fledged representatives of relevant ministries and governmental and non-governmental organizations and health professionals formed the National Committee for Tobacco Control in the general directorate of environmental and occupational health under the supervision of the Deputy Minister of Health. Several attempts failed to formulate tobacco use prevention programs and tobacco control policies in the first half of the 1991s. As a result, the plan was formulated, entitled “how to decrease smoking”, and presented to Parliament. The Islamic Consultative Assembly approved this plan. However, the Guardian Council rejected the plan as unconstitutional due to its financial burden on the government. This law was never implemented, despite the emphasis and stipulation that it must be enforced for all institutions. In 1997, the Council of Ministers approved the regulations relating to the ban on smoking cigarettes and other tobacco products in public places”; but this resolution was not implemented due to the resistance of specific sectors [21]. The content of the regulations were the ban on advertising of the tobacco industry, attaching warning messages on cigarette packs and banning the use and supply of cigarettes and other tobacco products in public places.

#### Existence of a comprehensive plan for tobacco control

Tobacco control advocates accepted the comprehensive plan to national control and campaign against tobacco, and the basis and provisions of which were based on FCTC and global evidence. NGOs and the MoHME were involved in compiling those as the Parliamentary Health Commission. Several meetings were held with the relevant executive bodies and were placed in various parliamentary commissions to increase the acceptance of this plan. Consultations were held between the speaker and members of parliament to gain advocates and make it acceptable to imitators. Also, during integrating the plan and the bill, the comparative and expert review was entrusted to the Parliamentary Research Center. The center organized meetings with relevant officials, institutions, grassroots organizations, and experts to increase its acceptability and applicability.

#### Pressures, requirements, treaties, agreements and international law

##### Framework convention on tobacco control (FCTC)

In 2003, Iran signed the WHO FCTC, considered tobacco control a public health priority, and ratified it in November 2005. In 2006, WHO FCTC was implemented in Iran [20]. This convention encourages countries to implement operational plans, including prevention of direct and indirect tobacco advertising, increasing tobacco tax, promoting smoke-free public places, attaching health warning labels to tobacco packaging. This convention played a vital role in the agenda-setting of the tobacco control policy-making process in Iran.

The 2011 United Nations Policy Declaration on the Prevention and Control of Non-communicable Diseases (NCDs) is a milestone in overcoming non-communicable diseases [22]. Officials have officially stated that NCDs pose a severe threat to health, the economy and society, and therefore have put its control over their agenda-setting.

##### Sustainable development goals 2030

In 2015, implementing the FCTC was reflected in the sustainable development goals (SDGs) 2030 in Objective 3. a, which is: “Strengthen the implementation of the FCTC in all member countries as appropriate”, and the indicator designated as the monitoring index and the WHO was determined as responsible for it [23].

##### International organizations

International organizations such as the WHO played a prominent role in the global governance of tobacco control. The WHO is involved in all stages of the policy-making process, including the tobacco control agenda, requiring member states to implement FCTC and develop guidelines, technical advice and financial support, and dissemination of tobacco control knowledge. The United Nations also plays a role in global tobacco control policy by global agenda-setting to prevent and control NCDs, the Millennium Development Goals and the SDGs. The World Bank also plays a role in Iran by advocating for tobacco tax increases, conducting research and advising and guidance and technical assistance on tobacco taxation in Iran.

### Political stream

#### Requiring the expediency council to the government to prepare a bill

In 1992, the Expediency Council ordered the government to prepare and submit a bill to reduce tobacco consumption to the parliament. The Islamic Consultative Assembly approved the proposal “how to gradually reduce and eliminate tobacco use” in 1991. However, the Guardian Council found it contrary to principle 75 of the Constitution of the Islamic Republic of Iran. It was discussed at the meeting of the Expediency Council, and the Expediency Council did not approve the amendment to the proposal. Therefore, this assembly, while approving the opinion of the Guardian Council, expressed that:

*“The government should investigate how to reduce tobacco use , by its authority, should directly take the relevant executive measures or prepare a proposed bill and submit it to the Islamic Consultative Assembly”* [24]*.*

However, the interviewees stated that 10 years have passed since such a requirement, and failure to prepare a bill by the government caused them to prepare the proposal by representatives of the Islamic Consultative Assembly and tobacco control advocates.

#### Entering into an international commitment: ratification of the FCTC in the Islamic consultative assembly

In this study, although the political stream had begun to a minimal extent years ago, it crystallized in 2003; When the FCTC, the world’s first evidence-based tobacco control public health treaty, opened in June. This historic achievement meant that it legally required the signatories to deal with tobacco use. After signing the convention in 2003, Iran approved the law on Iran’s accession to the FCTC in 2005 in the Islamic Consultative Assembly. Therefore, Iran joined the FCTC in 2005 and had to implement it. Many of the interviewees stated that Iran’s accession to the FCTC was one of the most important factors influencing the political process, creating political support and commitment regarding tobacco control. WHO FCTC served as a driving force for the adoption and implementation of the Comprehensive Tobacco Control Act after several previous unsuccessful attempts before WHO FCTC ratification.

According to this convention, the member states were committed to implementing comprehensive national strategies for tobacco control. Advocates of tobacco control also used such a requirement to gain support for tobacco control policies. Therefore, following Iran’s accession to the member states of the FCTC, Iran developed a comprehensive law on national tobacco control while actively participating in the convention of this treaty, by paragraph 1b of Article 1, through the national committee for tobacco control.

*“The MoHME, as the responsible organization for tobacco control subject to Article (5) of the Convention, must formulate and implement national, comprehensive strategies for tobacco control, and monitor them periodically to achieve this goal in coordination with the executive apparatus, implement training and implementation programs to reduce tobacco consumption. This Ministry will draft the bills and regulations to achieve the convention’s objectives and submit them to the relevant authorities”* [25]*.*

*“Each Party should develop, implement, periodically update and review comprehensive multisectoral national tobacco control strategies plans and programs by this convention and the protocols”* [26].

### Opening the opportunity window and joining of all three streams

Most of the interviewees believed that Iran’s access to the FCTC in the political process was the reason for opening the opportunity window. The policy window opened when all three streams had already been developed (Fig. 1). The problem stream was smoking, while the policy stream was a comprehensive plan for national control of tobacco. Members of parliament negotiations presented it and agreements were reached by the advocates of tobacco control and many of the country’s executive apparatuses, and the political climate was prepared for change. In the political stream, Iran undertook to adopt a comprehensive law in this field by signing the FCTC through Iran’s representatives and ratifying Iran’s membership at the convention in 2005. Thus, the three streams were joined, and a policy window opened. The adoption of the Comprehensive Law on Tobacco Control in the Islamic Consultative Assembly in 2006 [27] is considered a turning point in tobacco control activities in Iran.

*“But what happened in the world coincided with the bill that was to be drafted by the proposal that the deputies made. The WHO wrote the first international tobacco control treaty based on evidence obtained in various countries. The ministers of health of 192 countries ratified this agreement after five years of expert work and various meetings at the World Assembly of the Health Organization. Fortunately, our representative in Geneva was one of the 20 countries that signed this agreement”* (Participant. 33).

### Continuity of policy development

After the law’s approval, the national headquarters of control and campaign against tobacco products was established. The executive by-law of this law was developed in 2007 [28]. Before the privatization of the Iranian tobacco company in 2013, attending tobacco industry representatives in policy meetings as a government body was discontinued to ensure compliance with Article 5.3 of the Convention.

While a smoking ban in public places existed before the WHO FCTC, public places were not clearly defined. Two years after ratification, in 2007, Iran implemented a by-law banning tobacco product consumption such as smoking in all public places, workplaces, public transport and outdoor public spaces. In these years, different interpretations of the term “public places” caused the court of administrative justice to rule three times in favour of not offering hookahs in teahouses and traditional restaurants and to annul the approvals of the Council of Ministers and the President. Over 6000 environmental health inspectors were trained to enforce the law and for reporting violations of the bans. In 2007, all direct and indirect forms of tobacco advertising, promotion and sponsorship (TAPS) were prohibited. In 2009, pictorial health warnings on cigarette and tobacco product packaging were implemented [29].

Iran’s MoHME has developed and implemented a wide range of anti-tobacco mass media campaigns collaborating with relevant agencies, including a focus on hookah consumption, youth and females, to raise public awareness. Funding for tobacco control activities has been increased. In 2012, the Ministry of Interior banned the sale and use of e-cigarettes [30].

In 2015, Iran implemented a 14% tobacco tax increase in the budget. The total tax was only 21.7% of the retail price of the most widely sold brand of cigarettes [31], far shorter than 70% recommended [32].

Furthermore, global organizations played a significant role in policy development and implementation by providing FCTC Protocols. In 2014, Iran approved the law on Iran’s access to the WHO FCTC Protocol to eliminate illicit trade in tobacco products [33].

## Discussion

The tobacco control agenda-setting process in Iran was broadly consistent with Kingdon’s multiple-streams framework. As the findings showed, this process results from changes in three streams and the existence of political actors to link these streams and tap the opportunity window. These findings are consistent with studies conducted in different countries relevant to tobacco control [34–36]. In the present study, several factors facilitated the connection of streams and opened the opportunity window. Moreover, several factors facilitated the connection of flows and the opening of the opportunity window, including indicators on the severity of the problem, shaping the issue, feedback from past policies, the existence of a comprehensive proposal or national control of tobacco, the requirement of the Expediency Council to the government and the FCTC. In this study, the political stream of the FCTC had the most significant impact on the agenda-setting process. Various studies have also emphasized the importance of ratifying the FCTC as the first global evidence-based treaty on public health in different countries in creating an opportunity window and agenda-setting process [35, 37, 38]. In a study conducted in Turkey, tobacco control had become a political priority because of the development and convergence of multiple streams, including a fourth, a separate global stream. This global stream included the FCTC, the Bloomberg Initiative for tobacco control, and the global tobacco control network. In the Turkish study, we observed the importance of foreign policy in the evolution of the political stream. The country’s desire to join the European Union led to creating a political environment that embraced global norms for tobacco control and helped open the window of opportunity [35].

In our study, the role of policy entrepreneurs in linking streams and using the policy window was crucial. These policy entrepreneurs were in tobacco-related NGOs, members of the National Tobacco Control Headquarters at the MoHME, and the Islamic Consultative Assembly members. Other studies have pointed to the leadership of key informants and their role in advocacy and leadership change [14, 34–36, 39–42]. The motivations behind the actions of these individuals and key leaders go back to their interests, experiences, expertise, values, beliefs, and political ideologies in the studies included [14, 34, 36, 40, 42]. In Turkey, for example, high-ranking politicians, including former Prime Minister and current President, Recep Tayyip Erdogan, Dr. Recep Adag, former Minister of Health, and Dr. Erdol, Chairman of the Parliamentary Health Commission, also Erdogan’s physician, were all non-smokers interested in tobacco control.

The present study results showed that contrary to Kingdon’s theory that the three-stream streams are independent of each other [6], these three-streams had influenced each other. Thus, the political stream (FCTC) had strengthened the political stream (Comprehensive Plan for the Control and Control of Tobacco), and on the other hand, the strengthening of the policy stream cause further strengthened the political stream [35, 43].

The policy context has had a significant impact on the policy process of tobacco control in Iran. The influence of religious factors was an influence on tobacco control policy [20].

The most important international factors in the country influencing tobacco control policy were: access to the FCTC, protocol on the elimination of illicit trade in tobacco products, united nations policy declaration on the prevention and control of noncommunicable diseases 2011, sustainable development goals 2030, international organizations, international sanctions against Iran, international exploitation, international foreign media, modernization and globalization, and tobacco control experiences from other countries. These factors influenced both the agenda-setting and the policy solution [35]. Global agenda-setting influenced the agenda-setting in different countries. The role of contributions from international organizations, such as the WHO, has been mentioned in some studies. Their role in the studies included the publication of problem reports [35], technical assistance [35, 38], and financial assistance to tobacco control activities [35]. They have also been influential in the policy-making process in several studies by transnational tobacco companies [35, 38]. In several studies, the requirements related to before and after joining the unions and global treaties were influential in the agenda-setting [35, 38, 39, 44]. For example, Turkey’s strong desire to join the European Union created a political environment. Turkey has adopted global tobacco control norms to comply with the standards and norms of the European Union, Turkey has adopted global tobacco control norms [35]. Various studies have emphasized the importance of ratifying the WHO FCTC as the first evidence-based global public health treaty in various countries to create a window of opportunity for policy development [35, 37, 38].

### Limitations of study

The present study has some limitations. The first limitation was the problem of recalling information about processes and events related to past policy-making and legislative processes because of the long analysis period of some interviewees. In these cases, we tried to resolve this issue by using documents and reviewing the findings with several experts in this field. The second limitation was the issue’s sensitivity, which prevented stakeholders from engaging effectively and seriously in interviews. The researcher tried to increase the participants’ trust in the importance and application of research, the confidentiality of the information and the non-recording of audio to solve these problems. The following limitation refers to Kingdon’s framework, which originated in developed countries and has not been adapted to developing countries’ social and political context. As for the Kingdon’s framework, the results showed it needs to be adjusted and contextualized. The next constraint is related to exploring the unclear effect of entrepreneurs on the opening of policy windows and the ambiguity between them. Therefore, researchers use MSF to identify agenda-setting, especially in the health sector, and reduce potential limitations by combining it with other theories and models.

## Conclusion

The tobacco control agenda-setting process in Iran was broadly consistent with MSF. The present study results showed that contrary to Kingdon’s theory that the three-stream are independent, this three-stream had influenced each other. Thus, FCTC as a policy stream had strengthened the comprehensive plan for controlling tobacco as a political stream. On the other hand, the strengthening of the policy stream caused to strengthen the political stream further. However, there were several challenges in developing effective policies for tobacco control in the Iranian context.

In the problem stream, tobacco control actors need to ensure that credible indicators are available, providing evidence of the severity of the problem to policymakers, decision-makers, and the public. Also, the problem of tobacco should be raised as an issue that needs immediate attention and action. Regarding the policy stream, tobacco control advocates must form a solid coalition to reach a collective agreement on solving the problem. Therefore, evidence-based recommendations from global organizations such as the WHO can facilitate accepting solutions among stakeholders. In the political stream, although actors may not be able to directly influence the change of government or their different commitment to tobacco control or change of national mood, they must learn to recognize when the political environment is ready for change and prepare themselves to influence influential people in this field. Integrating health diplomacy into tobacco control in the country’s foreign policy as a political stream can also strengthen international cooperation and join groups working on the issue in other countries. It will improve the use of international experiences in the country. In terms of joining the three streams, the actors must prepare themselves for the policy window to open to include their favourite issues on the agenda. Therefore, we recommend paying attention to the process of policy change based on streams and making evidence-based decisions.

## Data Availability

All data produced or synthesized in this study are included in this published article.
